# Lectures on Physiology

**Published:** 1898-06

**Authors:** 


**Lectures on Physiology.** First Series.—*On Animal Electricity.* By AUGUSTUS D. WALLER, M.D., F.R.S. Pp. viii., 144. London: Longmans, Green, and Co. 1897.

In these lectures the method of experimenting on frog's and mammalian nerve and the graphic record of the results are dealt with in a clear and interesting manner. Professor Waller points out how infallibly the galvanometer responds to the condition of the nerve, whereas the contraction of muscle does not always express this faithfully. Using the photographic record of the galvanometer, he details most instructive experiments to show the effect of carbonic acid gas, alcohol, tobacco smoke, etc. on the vitality of the nerve. He shows that nerve is peculiarly sensitive to $CO_2$, which favours and facilitates nerve action, and suggests that the $CO_2$ formed during the disintegration of nerve-matter may possibly be reinvolved in some combination which may be stored up for future use, *e.g.* the nerve-fat.

Teachers and students of physiology should be thankful to Dr. Waller for his clever demonstration of polarisation currents and for his lucid explanation (aided by a good diagram on p. 109) of Matteucci's doctrine that these currents are essentially electrolytic. Except in lectures v. and vi., where we are brought into deep waters, everything in this small book is made quite clear to the careful reader; and this is high praise when we consider the intricate nature of the problems involved and the difficulty of experimenting.

